# Taking Impressions of the Mouth

**Published:** 1895-06

**Authors:** 


TAKING IMPRESSIONS OF THE MOUTH.—By James W. White, M. D., D. D. S. Publishers: S. S. White Dental Manufacturing Co., Philadelphia. 1895.

A second and enlarged edition of this valuable work has been published, and its contents comprise information of the most useful kind relating to the importance of a correct impression, conditions of the mouth, selection of the tray, and impression material, nature and manipulation of the different substances employed as impression materials, preparation of the mouth, special cases, partial cases, etc., etc. No treatise on this subject is as complete as this work of the late Dr. White, and the present revision has added greatly to its value.

